# Lower Pain Intensity Is Associated with the Use of Recreational Edible Cannabis Products Containing Delta-9-Tetrahydrocannabinol: A Secondary Analysis in Adults Self-Managing Their Chronic Low Back Pain

**DOI:** 10.3390/biomedicines14071642

**Published:** 2026-07-21

**Authors:** Jonathon K. Lisano, Luiza Rosa, Carillon J. Skrzynski, Angela D. Bryan, L. Cinnamon Bidwell

**Affiliations:** 1 Institute of Cognitive Science, University of Colorado Boulder, Boulder, CO 80309, USA; lcb@colorado.edu; 2 Department of Psychology and Neuroscience, University of Colorado Boulder, Boulder, CO 80309, USA; luiza.rosa@colorado.edu (L.R.); cariskrzynski.conway@colorado.edu (C.J.S.); angela.bryan@colorado.edu (A.D.B.)

**Keywords:** THC, CBD, cannabidiol, chronic pain, cannabis, marijuana, edible, naturalistic

## Abstract

**Background/Objectives:** Current literature regarding the efficacy of cannabis to reduce chronic pain intensity is mixed. Despite growing accessibility throughout the U.S., it is still unclear if the naturalistic use of recreational cannabis edible products is associated with decreases in pain intensity on days of use or longitudinally, and if these associations are dependent on the doses of delta-9-tetrahydrocannabinol (THC) and cannabidiol (CBD) being consumed. **Methods:** The trial (NCT03522324) from which these data were pulled was pre-registered in April 2018. Participants (N = 243; 56% female; mean age = 46 ± 12 years) with self-reported chronic low-back pain selected a recreational edible cannabis product to use exclusively, *ad libitum*, for 14 days. Labeled THC and CBD potency were used to determine product group: CBD-dominant (n = 97), THC + CBD (n = 112), or THC-dominant (n = 34). Participants completed daily surveys indicating current pain intensity (PROMIS; 0–10 scale), use or non-use of their product, and cannabinoid dose (THC and CBD). **Results:** Linear mixed effects showed a significant use × group interaction (*p* = 0.002), indicating that pain intensity was significantly lower on days of use in THC-dominant (*b* = −0.66, 95% *CI* [−0.94,−0.37]) and THC + CBD (*b* = −0.41, 95% *CI* [−0.57,−0.25]) groups compared to days when cannabis was not used. A significant group × time interaction (*p* = 0.02) indicated that pain intensity significantly decreased from day 1 to day 14 in those using THC + CBD products (*b* = −0.05, 95% *CI* [−0.07,−0.03]), with 36.6% of participants in the THC + CBD group experiencing ≥30% reduction in pain intensity from day 1 to day 14. Increasing doses of THC (*b* = −0.02, 95% *CI* [−0.04, −0.01]), not CBD (*b* = 0.003, 95% *CI* [−0.03, 0.01]), were associated with significantly lower pain intensity on days following product use, with increasing doses of CBD diminishing the impact of THC dose (*b* = 0.02, 95% *CI* [0.01, 0.04]). **Conclusions:** These findings indicate a complex relationship between THC, CBD, and pain intensity associated with the naturalistic use of recreational cannabis edible products. Lower daily pain intensity was associated with the use of products containing THC; however, dose models indicate that this association may be attenuated at higher doses of CBD. Additionally, only products containing relatively equal amounts of THC and CBD were associated with lower pain intensity after 14 days of observation.

## 1. Introduction

As of 2023, chronic pain affects more than 24% of adults in the United States [1]. Chronic low back pain is the most frequently reported type of chronic pain, impacting 41% [2]. In addition to a high rate of incidence, chronic low back pain is estimated to have a socioeconomic impact of $40 billion (USD) per year, and the incidence and burden are anticipated to increase as the population ages [3]. Due to the prevalence of chronic low back pain and the lack of effective treatment options, it is unsurprising that more than 85% of medical cannabis patients endorse pain as a motive for use [4,5].

Access to medicinal and recreational cannabis has been rapidly expanding throughout the U.S. for more than a decade. The effects produced by cannabis are in large part due to the presence of cannabinoids like delta-9-tetrahydrocannabinol (THC) and cannabidiol (CBD). While research into the therapeutic effects of cannabis may be lacking in some areas, the National Academy of Sciences states that there is substantial evidence for the efficacy of cannabis to treat chronic pain (Conclusion 4-1) [6]. In fact, a recent meta-analysis suggests that cannabis may provide similar reductions in pain and relatively fewer discontinuations due to adverse events compared to opioids [7]. A systematic review by Bell et al., 2025 also concluded that there were moderate benefits associated with the use of cannabis-based medications [8]. The most promising data for cannabis to reduce chronic pain comes from studies on nabiximols, a cannabis-based oral-mucosal spray which contains relatively equal doses of THC and CBD. A meta-analysis of randomized controlled trials assessing the medical indications of cannabinoid-based medications reported that, relative to placebo, nabiximols showed moderate evidence for improving chronic pain [9]. The effects of nabiximols on pain may be due to the presence of both THC and CBD, and their ability to interact with the diversely expressed endocannabinoid system that is present in the central nervous system, peripheral nervous system, and the immune system [10,11]. Nabiximols is not currently approved for medical use inside the U.S. As a result, more research is needed to explore the effects of cannabis on chronic pain using products readily available on the open market.

Cannabis products come in a variety of forms, including but not limited to inhalable (flower or vape), edibles, and topicals. Research shows that more than 93% of patients using cannabis for pain endorse using multiple routes of administration to manage their symptoms [12]. The most commonly endorsed routes of cannabis administration are inhaled cannabis and edibles [12], and there are distinct pharmacokinetic differences between these routes of administration. Compared to inhaled cannabis, orally ingested cannabis takes longer to reach maximum concentration and has a lower *C*_max_, but its active effects persist significantly longer [13]. In fact, orally ingested cannabis has a similar pharmacokinetic profile to nabiximols, with both having similar time to maximal concentration and area under the curve, except orally ingested cannabis had significantly higher *C*_max_ for both THC and CBD [14]. This suggests that the substantially longer period of active effects associated with cannabis edible ingestion could offer similar therapeutic potential to that of nabiximols in patients with chronic pain.

Despite a similar pharmacokinetic profile to nabiximols, the current literature regarding the impact of orally ingested cannabis on chronic pain is mixed and is based largely on clinical samples. For example, in patients with fibromyalgia, a double-blind placebo-controlled trial reported significant improvement in pain intensity following 8 weeks of standardized orally ingested THC-dominant cannabis oil [15]. Conversely, in patients with osteoarthritis or psoriatic arthritis, a double-blind placebo-controlled trial of orally ingested CBD-dominant cannabis did not observe significant improvement in pain intensity after 12 weeks of standardized use [16]. There is limited evidence as to how the naturalistic use of recreationally available cannabis edible products impacts pain intensity in non-clinical populations with self-reported chronic pain. A study from our research group observed that two weeks of naturalistic edible cannabis was associated with changes in pain intensity, and these changes may differ based on cannabinoid (THC vs. CBD) and use context (extended two-week effects vs. an acute use setting) [17]. In this study, products containing THC were associated with acute reductions in pain intensity, while decreases in pain intensity over two weeks were associated with more frequent use of CBD-dominant products [17]. These data suggest that there may be variations between clinical and non-clinical samples, and improvements in pain intensity may vary based on product type and use context. Previous findings are limited by the collection of data at discrete time points over weeks or months, and have not captured day-to-day variations in natural use (i.e., use or non-use of cannabis, frequency of use, and THC/CBD dose) that would be reflective of the natural daily use habits of those with access to legal cannabis.

The present secondary analysis focuses on addressing this literature gap by leveraging daily data from the previously mentioned publication by Melendez et al. (2024) that explored the longitudinal and acute effects of edible cannabis on chronic pain intensity [17]. Using previously unreported daily self-report surveys, the present analyses explored the nuanced relationships between edible cannabis use and pain intensity at the daily level. Participants were those seeking to initiate cannabis to reduce their chronic low back pain. These participants self-selected an edible cannabis product to use *ad libitum* over a 14-day observational period. To assess if associations observed in the primary models were due to variations in product use, a preliminary analysis was done to assess if the number of times participants used their products, on use days, varied by group or across time. The first aim of the primary analyses was to determine if pain intensity was lower on days on which participants used their product compared to days on which they did not use it, and if these effects were dependent on the product’s chemovar (i.e., CBD-dominant, THC-dominant, or THC + CBD). The second aim was to assess whether changes in pain were impacted by the number of times participants used their product in a day and if this was chemovar dependent. Finally, an exploratory aim assessed the association between daily THC and CBD dose on daily pain intensity. Based on previous research, we predicted that reductions in pain intensity at the daily level would be associated with the use of THC-dominant or THC + CBD products, and more pronounced reductions in pain intensity would be associated with higher frequency of use.

## 2. Materials & Methods

### 2.1. Participants

**Figure 1:**
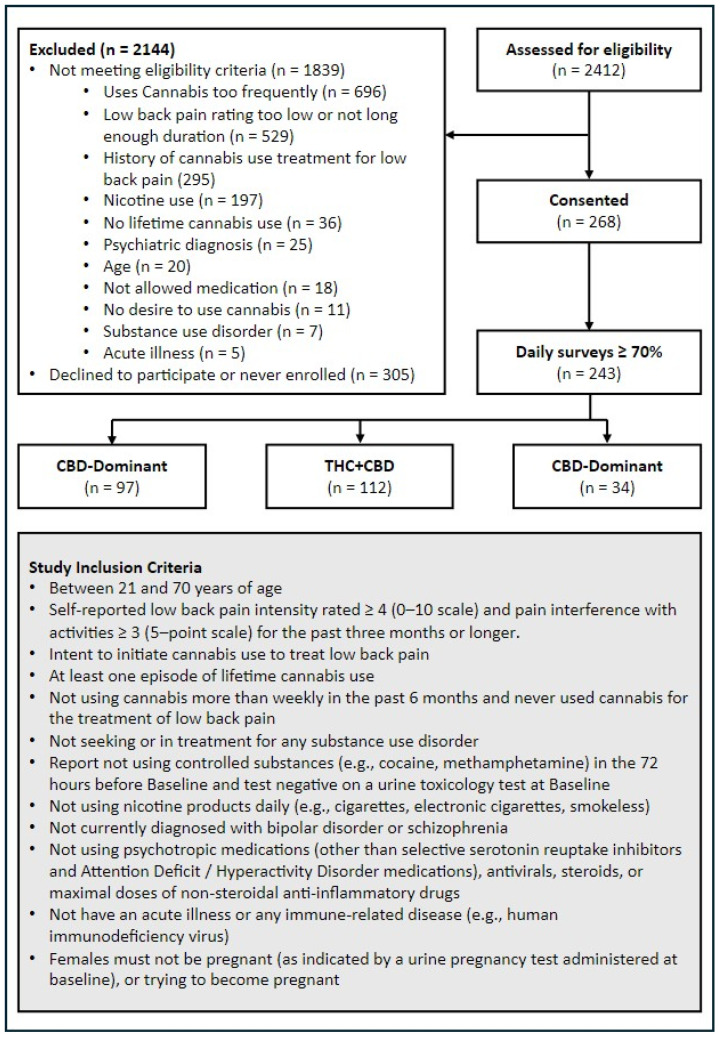
Consort and Study Inclusion Criteria.

To be eligible for study participation, participants had to be between the ages of 21–70 years of age, have previously used cannabis at least once in their life, and intend to initiate cannabis use to treat their self-reported, chronic low back pain. Furthermore, they had to self-report having experienced low back pain for ≥3 months, have low back pain intensity of ≥4 (0–10 scale), and have pain interference with activity ≥3 (5-point scale) [18,19]. A full participant consort and list of study inclusion and exclusion criteria can be found in Figure 1. Participants were recruited from the Denver/Boulder area through social media, mailed and posted flyers, and community events. The study was approved by the University of Colorado Boulder’s Institutional Review Board (IRB) and followed all ethical standards for the 2008 revision of the Helsinki Declaration. The parent study was pre-registered on 17 April 2018 at Clinicaltrials.gov (NCT03522324).

### 2.2. Timeline

After screening, participants attended a baseline in-person study visit at our research laboratory located on the university’s campus. During this baseline visit, participants completed informed consent, demographic measures, and surveys, and were oriented to the virtual daily automated surveys. Beginning on the day after the baseline visit, daily automated surveys were sent to participants via the research electronic data capture management (RedCap) platform for 14 days [20]. The daily surveys contained questions regarding the use of their selected study product and their current low back pain intensity. Up to $80 in compensation was provided to participants for completing the aspects of the study utilized for analysis here: $60 for completion of the baseline visit, and $1 per day for each of the daily surveys they completed over the 14-day *ad libitum* period. A bonus of $6 was awarded if participants completed at least 12 of the 14 daily surveys.

### 2.3. Cannabis Product Selection

At the baseline visit, participants received standardized information about locally available cannabis edible products. This included safety guidelines for edible cannabis use based on state public health resources (e.g., “start low and go slow”), as well as pricing information and where products could be purchased. Participants were instructed to buy as much or as little of their chosen product as they anticipated needing over the following 14 days. They then visited a dispensary of their choice and independently selected an edible cannabis product for managing their chronic low back pain. In line with the study’s naturalistic, *ad libitum* design, participants were instructed to use the product as frequently as they felt necessary to address their individual needs. Participants were not reimbursed for the purchase of their product, and were instructed to refrain from using any other cannabis products, outside of their chosen study product, for the duration of the study. Dispensaries were not involved in the study’s funding, design, analysis, or dissemination.

Per Colorado state regulations, all recreational cannabis products must undergo independent laboratory testing by facilities accredited by the International Organization for Standardization (ISO) 17025 standards [21]. These tests are in accordance with state-mandated product labeling, which includes THC and CBD concentrations. After purchasing their selected product, participants uploaded a photo of the product label to REDCap [20]. To maintain blinding of the primary research team, these photos were reviewed by a research associate who was not involved with in-person data collection. Labeled THC and CBD concentrations were extracted from these photos and used for subsequent product chemovar classification, as described below.

Note. Due to Colorado state regulations requiring cannabinoid concentrations to be disclosed on all cannabis product labels, and because participants were allowed to self-select their cannabis edible products, participants were not blinded to the cannabinoid content (THC and CBD) of the product they used over the 14 days of the study.

### 2.4. Edible Cannabis Chemovar Stratification

Participant-selected products were categorized into three chemovar groups based on their labeled THC and CBD content (reported in milligrams) [17]. Products classified as CBD-dominant had five times more CBD compared to THC, and THC-dominant products had five times more THC compared to CBD. Products that did not meet the aforementioned thresholds for either chemovar group were categorized as THC + CBD products.

### 2.5. Baseline Measures

Participant demographic information regarding age, gender, education (bachelor’s degree or higher vs. associate’s degree or lower), and race was collected.

### 2.6. Pain Interference

The Roland Morris Disability Questionnaire (RMDQ) [22] is a self-report measure used to assess the severity with which an individual’s low back pain has interfered with their daily life over the past two weeks. This measure contains 24 items (α = 0.83), with each item producing a cue like “Because of the pain in my back, I lie down to rest more often” and “I have trouble putting on my socks (or stockings) because of the pain in my back”. The number of responses that participants have experienced is then summed to create a total score.

### 2.7. Pain Intensity

Participants’ current, average, and worst low back pain intensity over the past 7 days were assessed using the Pain Intensity Short Form [23]. Ratings for these items ranged from “0” indicating “no pain” to “10” indicating “worst imaginable pain”.

### 2.8. Cannabis Pain Expectancy

Participants’ expectations that cannabis would reduce pain were assessed using a single item from the Impact of Marijuana on Pain (IMP) for “Decreased Pain” in response to the question, “Which of the following benefits do you expect to get from cannabis? (Please select the level of change you expect)”. Response options were “No improvement at all”, “Not very improved”, “Somewhat improved”, and “Very improved.” Responses were coded on a 0–3 scale, with 0 = “No improvement at all” and 3 = “Very improved.”

### 2.9. Substance Use

Cannabis Use Disorder. Symptoms of cannabis use disorder (CUD) over the past 12 months were measured using the DSM-5 modified Marijuana Dependence Scale [24]. The scale includes 11 items (α = 0.69), with the score reflecting the total number of symptoms reported. Scores range from 0–11.

Cannabis and Alcohol Use. The frequency (days of use) of cannabis and alcohol use over the previous 14 days was measured via the Online Timeline Follow-back assessment (O-TLFB) [25].

Study product use. Daily surveys (described below) were used to calculate the number of days that participants used their chosen study product over the 14-day study period, the total number of times that participants used their product over the study, the average number of times participants used their products on days of use, and average THC and CBD dose (reported in milligrams (mg)).

### 2.10. Daily Measures

Daily automated surveys asked participants to self-report their current pain intensity, whether or not they had used their product, the number of times they had used their product, and cannabinoid dose (estimated THC and CBD in mg).

### 2.11. Current Pain Intensity

Using a single item from the Pain Intensity Short Form [23], participants self-reported their current pain intensity in response to the question, “What is your level of lower back pain currently (in which 0 represents no pain and 10 represents the worst imaginable pain)?”.

### 2.12. Product Use

Daily use status. Participants responded yes or no to the question, “Did you choose to use your edible product in the past 24 h?”. Responses were binary coded, with “No” indicating no product use (0) and “Yes” indicating they had used their product (1).

Number of product uses in the past 24 h. If participants responded “Yes” to having used their study product in the past 24 h, they were then asked the following question: “How many separate periods of time did you use your edible product in the past 24 h?”. Response options to this question ranged from “1” (indicating they had used their product once in the past 24 h) to “10” (indicating they had used their product 10 or more times in the past 24 h).

Cannabinoid Dose. If participants responded “Yes” to having used their study product in the past 24 h, they were also asked to report the total dose of THC and CBD they had used in the past 24 h. The following questions were used: “How many total THC milligrams (best guess) did you use/consume (e.g., eat) in the past 24 h?” and “How many total CBD milligrams (best guess) did you use/consume (e.g., eat) in the past 24 h?”. Responses were recorded in mg and were reported using a drop-down menu with options ranging from “1 mg” to “200 or more mg”.

### 2.13. Statistical Analysis

Analyses were limited to participants who had completed at least 70% of their daily surveys over the 14 days. All analyses were performed in R [26] utilizing the dplyr [27], sjPlot [28], ltm [29], nlme [30], and emmeans [31] libraries. Figures were generated using the ggplot2 library [32]. Analyses of Variance (ANOVAs) were used to assess group differences in baseline descriptive characteristics for age, pain intensity (current, average, and worst), cannabis pain expectancy, CUD symptoms, and frequency of substance use prior to study start (cannabis and alcohol). Chi-square analyses assessed group differences in gender, education, and race. ANOVAs were also used to assess differences in basic study product use characteristics like days of use and total number of product uses over the study period.

An initial descriptive linear mixed effect model was run to determine if the number of times participants used their products, on use days, varied by group or over time. This was done to determine if associations observed in the primary models might be the result of differences in patterns of use. Separate linear mixed effects models were then run to assess 1. the association between cannabis use/no use in the past 24 h and pain intensity, 2. if the number of separate times participants used their products in the past 24 h was associated with changes in pain intensity, and 3. the associations between cannabinoid dose (milligrams of THC and CBD) and pain intensity on the *day of* and *the day following* product use (see below). All linear mixed effect models included random intercepts to account for subject-level clustering and per-participant autoregressive (order 1) error structures over time. For significant interactions, post hoc trend analyses were conducted using the emtrends function of the emmeans library. Where appropriate, between-group comparisons of these trends were done using the pairs function of the emmeans library. Due to the inability to blind participants, baseline cannabis pain expectancy was controlled for in all linear mixed-effect models. In addition, due to group differences at baseline (Table 1), participant age, education, ethnicity, and worst pain intensity were controlled for in all models utilizing product group as a predictor.

### 2.14. Frequency of Edible Use on Days of Use

To first understand if there were differences by product group with respect to the number of times participants used their products on days of use, or if this changed over the 14 days, a linear mixed effect model utilized predictors of product group and time on this outcome. To limit the analytical impact of days in which participants did not use their product, non-use days were dropped from the analysis.

### 2.15. Cannabis Use and Daily Pain Intensity

To explore the association between *day of* edible cannabis use and current pain intensity, a linear mixed effect model compared non-use days to use days (use status) by product group (CBD-dominant, THC + CBD, THC-dominant) over time (Day 1 to Day 14). This model included the main effects of use status, group, and time as well as all possible two and three-way interactions.

### 2.16. Number of Separate Uses per Day and Daily Pain Intensity

To assess if changes in pain intensity varied based on the number of separate times participants had used their product in the past 24 h, a linear mixed effect model utilized predictors of the number of separate times participants reported using their products in the past 24 h and product group. This model included the main effects for the number of separate product uses and group, as well as the number of separate uses × group interaction. To limit the analytical impact of days in which participants did not use their product, non-use days were dropped from the analysis.

### 2.17. THC and CBD Dose and Pain Intensity

To assess if there was a relationship between current pain intensity and the doses of THC and CBD consumed on the same day (i.e., are higher reported levels of pain associated with consuming higher doses on the same day?), the first linear mixed effect model assessed the relationship between participants’ current pain intensity and doses of THC and CBD consumed on *the day of* product use. Then, to assess if there was a relationship between the doses of THC and CBD consumed and pain intensity *the following day* (i.e., are higher doses used on the previous day associated with lower pain intensity the day following use?), a second linear mixed effect model was run with the doses of THC and CBD lagged to the following day. Each of these models included the main effects of THC and CBD dose, and the THC × CBD dose interaction.

## 3. Results

### 3.1. Descriptive Information

A total of 243 participants met study inclusion criteria and had at least a 70% completion rate of the daily surveys. Of the 243 participants, 97 self-selected into a CBD-Dominant product, 112 into a THC + CBD product, and 36 into a THC-Dominant product. Across the entire sample, the average duration of self-reported chronic low back pain was 10.8 years. At baseline, there were significant differences across product groups in participant age, education, and race (Table 1). There were no differences in baseline self-reported RMDQ pain interference scores or current and average pain intensity by product group, but there was a significant group difference in self-reported worst pain intensity at baseline (Table 1). Post-hoc analysis indicated that worst pain intensity was significantly higher in the THC + CBD group compared to the CBD-dominant group (*p* = 0.03). Full detailed participant characteristics can be found in Table 1.

At baseline, there were no differences in CUD scores by group or cannabis pain expectancy (Table 1). On average, participants were using cannabis products less than once per week before study enrollment (Table 1). Based on daily survey responses, participants used their chosen study product an average of 8.72 ± 3.81 days (range: 0–14) for a total of 11.88 ± 8.01 separate uses (range: 0–42) during the 14-day study period. This equated to an average of 1.37 ± 0.74 separate uses per day, on days of product use. Detailed descriptive data for study product use by product group can be found in Table 2.

### 3.2. Linear Mixed Effect Models

#### 3.2.1. Frequency of Edible Use on Days of Use

In the preliminary linear mixed effects model assessing the number of separate times that participants used their product on days of use, there were no significant main effects of group (*F*(2,238) = 2.58, *p* = 0.08) or time (*F*(1,1869) = 0.13, *p* = 0.71). Furthermore, no significant group-by-time interaction was observed (*F*(2,1869) = 0.52, *p* = 0.59). These findings indicate that on days of use, there were no significant differences in the frequency with which groups were using their product. Additionally, the number of times participants used their products on days of use stayed consistent throughout the study.

#### 3.2.2. Cannabis Use and Daily Pain Intensity

The linear mixed-effects model exploring the associations between *day of* edible cannabis use and current pain intensity showed no significant main effects of use status, group, or time on daily pain intensity (*p*’s > 0.05). However, two significant two-way interactions were observed: 1. use status × group (*F*(2,2917) = 6.13, *p* = 0.002) and 2. group × time (*F*(2,2917) = 3.79, *p* = 0.02).

**Figure 2:**
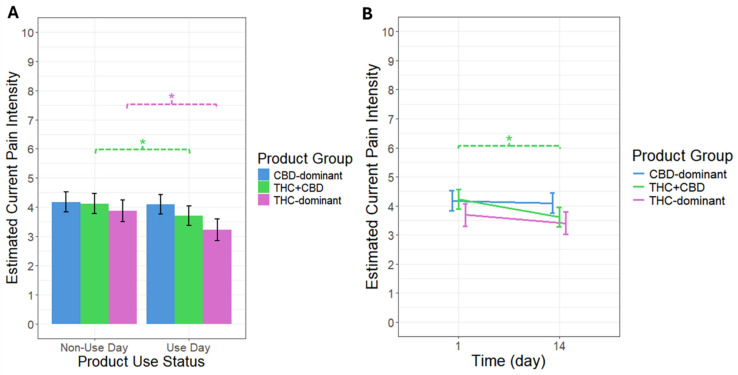
*Note.* Estimated means ± SE are plotted. (**A**) Use status was determined if participants reported using (Use Day) or not using (Non-Use Day) their chosen cannabis product in the past 24 h. There was no significant difference in current pain intensity if participants had or had not used CBD-dominant products in the past 24 h. * Current pain intensity was significantly lower in participants who had used THC-dominant or THC + CBD products in the past 24 h compared to days in which they had not used their products. (**B**) Over the 14-day study period, current pain intensity did not significantly change in participants using CBD-dominant or THC-dominant products. Current pain intensity significantly decreased from day 1 to day 14 in participants using THC + CBD products. * Indicates the slope of the estimated means significantly differs from zero.

When comparing pain intensity on days of use to non-use days, use of cannabis edible products was associated with significantly lower pain intensity in the THC-dominant (*b* = −0.66, SE = 0.14, 95% *CI* [−0.94, −0.37]) and THC + CBD (*b* = −0.41, SE = 0.08, 95% *CI* [−0.57, −0.25]) groups. There was no significant change in current pain intensity associated with the use of CBD-dominant edible products (*b* = −0.08, SE = 0.10, 95% *CI* [−0.28, 0.11]). Based on estimated means, average pain intensity was 1.9%, 9.9%, and 17.0% lower on days of cannabis use compared to days of no cannabis use in the CBD-dominant, THC + CBD, and THC-dominant groups, respectively. Estimated means for *same-day* pain intensity on product non-use and use days by group are presented in Figure 2A.

Regarding the significant group × time interaction, there were no significant changes in daily pain intensity from day 1 to day 14 in the THC-dominant (*b* = −0.02, SE = 0.02, 95% *CI* [−0.06, 0.01]) or CBD-dominant (b = −0.01, SE = 0.01, 95% *CI* [−0.03, 0.02]) groups. However, in the THC + CBD group, there was a significant decrease in daily pain intensity from day 1 to day 14 (b = −0.05, SE = 0.01, 95% *CI* [−0.07, −0.03]). Based on estimated means, average pain intensity was 1.9%, 14.4%, and 7.9% lower on day 14 compared to day 1 in the CBD-dominant, THC + CBD, and THC-dominant groups, respectively. Additionally, n = 26 (26.8%), n = 41 (36.6%), and n = 9 (26.5%) participants experienced ≥30% reduction in self-reported pain intensity from day 1 to day 14 in the CBD-dominant, THC + CBD, and THC-dominant groups, respectively. Estimated means for daily pain intensity by group over time are presented in Figure 2B.

Given the unbalanced nature of the cannabis product groups, findings should be interpreted with caution. However, sensitivity analysis excluding the THC-dominant group demonstrated that the use status × group (*F*(1,2502) = 6.33, *p* = 0.01) and the group × time (*F*(2,2502) = 7.48, *p* = 0.006) interactions remained significant, supporting the findings.

Note. Based on findings reported in the “Number of Separate Uses Per Day and Daily Pain Intensity” section of the results, two supplemental linear mixed effects models were run to assess whether the number of days participants used their study product during the 14 days of observation moderated the aforementioned use status × group and group × time interactions. Within these models, the significance of the use status × group (*F*(2,2917) = 4.77, *p* = 0.008) and group × time (*F*(2,2923) = 3.56, *p* = 0.03) interactions persisted, but there were no main effects for the number of days participants used their study product (*p*’s > 0.49), nor did the number of days participants used their study product moderate the use status × group (*F*(2,2917) = 0.65, *p* = 0.52) or group × time (*F*(2,2923) = 1.03, *p* = 0.36) interactions, suggesting that the number of days that participants used their study product over the 14 days did not impact the aforementioned daily or longitudinal relationships.

#### 3.2.3. Number of Separate Uses per Day and Daily Pain Intensity

**Figure 3:**
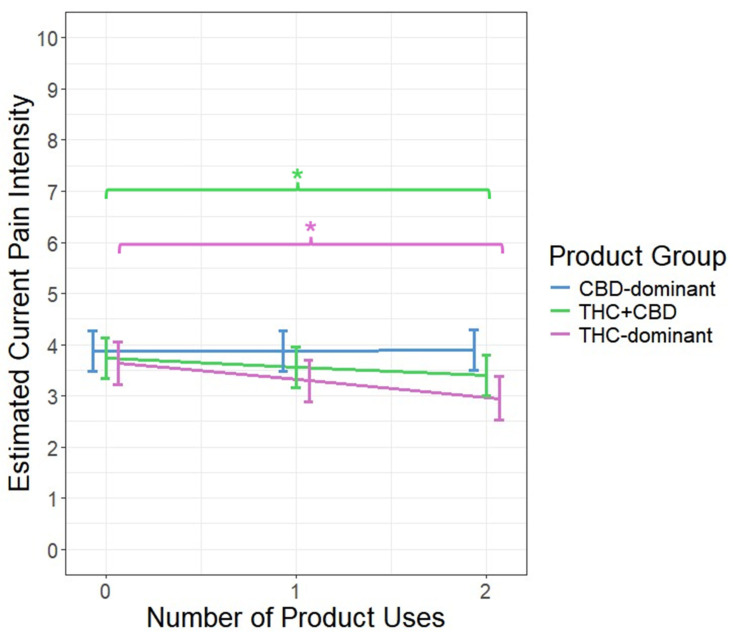
*Note.* Estimated means ± SE are plotted. If participants reported using their product in the past 24 h, they were asked how many times they had used their product over that time frame (Number of Product Uses). There was no significant change in current pain intensity regardless of the number of times participants in the CBD-dominant group used their product on days of use. Both the THC-dominant and THC + CBD groups observed significant decreases in current pain intensity as the number of times they used their product increased. Estimated means were calculated at 0, 1, and 2 product uses per day based on the overall average of 1.37 product uses per day, and 92.8% of participants reported using either once or twice on days of use. * Indicates the slope of the estimated means significantly differs from zero.

The linear mixed effects model assessing whether changes in pain intensity varied based on the number of separate times participants had used their product in the past 24 h showed a significant group × number of separate uses interaction (*F*(2,2916) = 6.13, *p* = 0.002). This interaction showed that increases in the number of times participants used their product over the past 24 h were associated with significant reductions in pain intensity, but only in the THC-dominant (*b* = −0.35, SE = 0.10, 95% *CI* [−0.54, −0.16]) and THC + CBD groups (*b* = −0.17, SE = 0.06, 95% *CI* [−0.28,−0.0–5]). There were no significant reductions in pain intensity, regardless of how many times participants used their product, in the CBD-dominant group (*b* = 0.01, SE = 0.05, 95% *CI* [−0.09, 0.11]). These findings indicate that more frequent product use throughout the day was associated with additional reductions in pain intensity, but only in the THC-dominant and THC + CBD groups. The estimated means showing the group × number of uses relationship are in Figure 3.

#### 3.2.4. THC and CBD Dose and Pain Intensity

**Figure 4:**
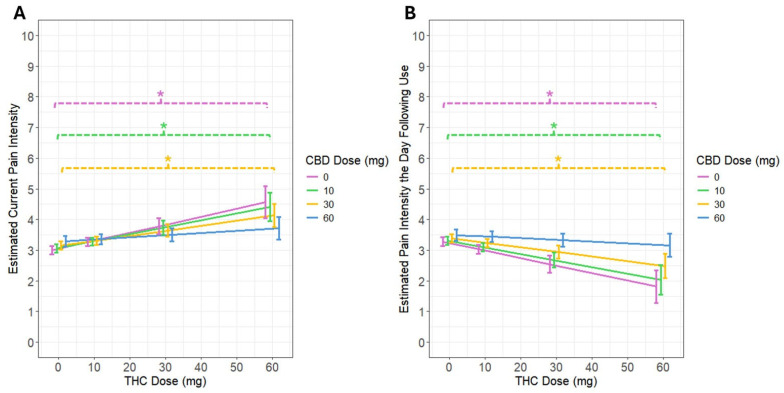
*Note.* Estimated means ± SE are plotted. THC and CBD doses were self-reported if participants had reported using their product in the past 24 h. (**A**) There was a significant positive relationship between the dose of THC consumed within the past 24 h and current pain intensity. There was no significant relationship between the dose of CBD consumed in the past 24 h and current pain intensity. The relationship between the dose of THC and pain intensity was mitigated with increasing doses of CBD. * Indicates the slope of the estimated means significantly differs from zero. (**B**) There was a significant negative relationship between the dose of THC consumed within the past 24 h and pain intensity the following day. There was no significant relationship between the dose of CBD consumed within the past 24 h and pain intensity the following day. The relationship between the dose of THC and pain intensity was mitigated with increasing doses of CBD. * Indicates the slope of the estimated means significantly differs from zero.

In the linear mixed effect model assessing the associations between THC and CBD doses the day of use and current pain intensity, a significant interaction between THC and CBD dose was observed (*F*(1,1532) = 4.16, *p* = 0.04). Tests of simple effects indicated that increasing doses of THC (with 0 mg CBD) were associated with higher reported current pain intensity (Figure 4A: *b* = 0.03, SE = 0.01, 95% *CI* [0.01, 0.04]). This association was not observed for consumed doses of CBD, regardless of the amount of THC participants had consumed (Figure 4A: *b* = 0.01, SE = 0.01, 95% *CI* [−0.01, 0.02]). Furthermore, the association between current pain intensity and the amount of THC consumed within the past 24 h is mitigated with increasing doses of CBD (Figure 4A).

In the linear mixed-effect model assessing the association between the doses of THC and CBD consumed on pain intensity *the following day*, there was again a significant two-way interaction between THC and CBD dose (*F*(1,1344) = 3.93, *p* = 0.04). Tests of simple effects tests indicated that increasing doses of THC (with 0 mg CBD) were associated with lower reported pain intensity *the following day* (Figure 4B: *b* = −0.02, SE = 0.01, 95% *CI* [−0.04, −0.01]). The effects of THC dose were mitigated as CBD dose increased (Figure 4B: *b* = 0.02, SE = 0.01, 95% *CI* [0.01, 0.04]). In contrast, increasing doses of CBD (with 0 mg THC) were not associated with changes in pain intensity the following day (Figure 4B: *b* = 0.003, SE = 0.003, 95% *CI* [−0.03, 0.01]).

## 4. Discussion

Building on the work of Melendez et al. 2024 [17], this study aimed to explore the impact of daily variations in naturalistic recreational edible cannabis use on pain intensity in participants with self-reported chronic low back pain and whether changes in pain depended on the cannabinoid content of the edible product being used. These data provide novel insight and demonstrate that on days on which participants reported having used their products within the past 24 h, they also reported significantly lower pain intensity compared to days in which they reported not using their products in the past 24 h. Further, as the number of separate times participants reported using their products in the previous 24 h increased, lower reported current pain intensities were observed. However, these findings were limited to the use of THC-dominant and THC + CBD products. Although the present study did not observe a statistically significant association between CBD use and pain intensity, regardless of dose or frequency of use, research in more robust samples is needed to determine if these associations persist. These results align with the predictions that daily and longitudinal reductions in pain intensity would be associated with the use of THC-dominant and THC + CBD products, and not CBD-dominant products.

Although statistically significant group-dependent differences in pain intensity were observed, it is important to discuss the clinical impact of the daily and longitudinal findings. On average, pain intensity was 10–17% lower on days in which participants used THC + CBD or THC-dominant products within the past 24 h. These daily reductions are below the traditional 30% clinical threshold for a moderately meaningful change in pain intensity and would fall into the minimally important (10–20% reduction) clinical category [33]. Similarly, on average, pain intensity was 14% lower on day 14 compared to day 1 in participants using THC + CBD edible cannabis products, which would also be considered a minimally important clinical change [33]. Although 36.6% of participants in the THC + CBD group experienced a ≥30% reduction in pain intensity from day 1 to day 14, it is difficult to compare the impact of this finding without a comparable placebo control. The lack of moderate meaningful reductions being observed could be the result of a variety of factors, including but not limited to differences in underlying physiological mechanisms, chronic pain etiology, or THC:CBD ratio and dose.

While reductions in pain intensity were observed in THC-dominant and THC + CBD products on the days of product use, longitudinal reductions in pain intensity were only observed in participants using THC + CBD products. This is the first naturalistic cannabis use study to report these associations at both the daily and longitudinal levels. These findings align with previous placebo-controlled clinical trials for nabiximols, and contribute further support for cannabis products containing both THC and CBD as a promising option for both daily and longitudinal chronic pain management. Although not directly measured in this study, it is plausible that the observed differences between daily and longitudinal changes in pain intensity are the result of different physiological mechanisms. We hypothesize that the daily associations may be the result of THC’s acute physiological effects, while the longitudinal effects may be the result of the immunomodulating effects of both THC and CBD [34,35,36]. These immunomodulatory effects have been observed in pre-clinical studies, but research has yet to establish the anti-inflammatory effects of cannabinoids as a potential mechanism for reductions in pain intensity in clinical samples. A large portion of chronic pain is believed to be the result of chronic inflammation [37], and THC and CBD have been observed to reduce inflammation better synergistically compared to individually [38]. Alternatively, there is a working theory that chronic pain alters synaptic plasticity. It is plausible that the observed associations in this study may be in part due to cannabinoids blunting the consolidation of pain memories by reducing activity-regulated, cytoskeleton-associated (Arc) expression via the CB1 receptor in the central nervous system [39,40]. Although the data surrounding this mechanism are limited, it is worth exploring how the potential neurological and psychological mechanisms of chronic pain are impacted by the use of cannabinoids.

Although the etiology of chronic pain is diverse and was not accounted for in the present analyses, the reductions in daily pain intensity associated with the use of THC-dominant and THC + CBD products may be due to THC’s effects on the endocannabinoid system, which is broadly expressed in both the central nervous system and the periphery [10,11]. Within the central nervous system, THC has been associated with reduced functional connectivity between the anterior cingulate and the sensory motor cortices, and reduced activity in pain-processing centers like the dorsolateral prefrontal cortex [41]. Peripherally, cannabinoid 1 (CB1) receptors have been observed to play a central role in the regulation of nociceptive pain signaling [42]. THC is an agonist of the CB1 receptor [43], whose activation reduces synaptic activity and signal transduction [44]. These alterations in central and peripheral pain processing via THC could, in part, explain the lower levels of pain reported not only on days in which participants used their products but also in the dose models observing associations of lower pain intensity with increasing THC dose on days following product use. Conversely, CBD acts as a negative allosteric modulator of the CB1 receptor that decreases the likelihood of THC binding to the CB1 receptor [45]. This difference in physiological mechanisms within the CNS could explain why CBD was not associated with daily reductions in pain intensity, and explain why THC-associated reductions in pain intensity appeared to be attenuated with increasing CBD in the dose model. More research is needed to determine the optimal doses and ratios of THC and CBD to observe daily and longitudinal reductions in pain intensity.

Many individuals see CBD as an attractive alternative therapy for mitigating chronic pain compared to THC. This may be the result of CBD not producing the intoxicating effects that are associated with THC. Yet, the use of CBD-dominant products and increasing doses of CBD were not associated with reductions in pain intensity in the present study. This may be the result of the relatively low self-administered doses of CBD that participants used. While some participants reported using CBD doses as high as 200 mg/day, the average dose of CBD consumed in the CBD-dominant product group was ~26 mg/day. The present findings align with a previous clinical trial that reported no changes in pain intensity relative to placebo associated with the daily use of 30 mg CBD softgels over 12 weeks [16]. However, other clinical data utilizing doses of CBD ranging from 100 to 300 mg/day for up to 13 weeks did report significantly lower pain relative to placebo [46,47]. Even though the aforementioned studies focused on samples outside of chronic low back pain, in the present study, it is plausible that the lack of significant changes in pain intensity with the use of CBD-dominant products is the result of the relatively low doses of CBD being used. Although clinical research on the analgesic effects of CBD at standardized doses is still limited, this study was designed to assess whether typical product doses sold on the legal market impact perceived pain intensity. Research should continue to explore the analgesic effects of CBD at higher, clinically relevant doses.

Strengths of this study are the use of a naturalistic *ad libitum* design examining the use of edible cannabis products purchased at legal recreational dispensaries, and the incorporation of novel daily data to assess the nuanced variations in daily pain intensity and edible cannabis use. This approach has high external validity and is reflective of real-world use, giving insight as to the effectiveness of self-selected edible cannabis to manage self-reported chronic pain intensity. Despite those strengths, participants were not randomized or blinded to their treatment, and even though cannabis expectancy effects were controlled for in our models, selection and expectancy bias still may have impacted these findings. These limitations largely result from the fact that randomized trials utilizing legal market cannabis are not permitted. Furthermore, the absence of both random assignment and a placebo control limits the causal interpretation of these findings. Due to state labeling requirements for cannabis, the present study was not able to blind participants to the product they were consuming. Compared to other clinical trials assessing the effectiveness of cannabis to reduce pain, the study observation period was rather short (14 days), and future research should determine these effects over longer durations. Further, the average participant was white and well educated, limiting the implications of these results to more diverse populations experiencing chronic pain. Future research should look to expand these findings in samples with greater diversity.

In Colorado, there have been reports of THC labeling inconsistencies in cannabis flower and concentrate products [48]. While there is limited data indicating that edible medical cannabis products from California, Washington, and Mississippi also demonstrate labeling inconsistencies [49,50], it is unclear if these inconsistencies extend to recreational edible products in Colorado. These potential labeling discrepancies should be taken into consideration in future studies using recreationally available cannabis products. It is also important to note that, while pragmatic, daily self-report doses of THC and CBD in this study remain susceptible to reporting error and recall bias. Findings from the present THC and CBD dose models should be interpreted with caution, and future research should look to explore these relationships in a more controlled manner. These findings are limited to general self-report of low back pain and are not specific to differences in low back pain etiology or pathology. Future research should assess if the associations reported in the present study differ by chronic pain etiology/pathology or location of the chronic pain (back, neck, extremities, etc.). It should also be considered if behavioral or biological processes related to chronic pain (i.e., mood or inflammation) play moderating or mediating roles in these associations. Finally, research should use objective measurement, such as actigraphy, to assess the interdependent effects of cannabis on pain, physical activity, and sleep quality.

## 5. Conclusions

Daily data collected over 14 days of naturalistic cannabis use demonstrated that reductions in participants’ current pain intensity were associated with the use of cannabis edible products, but only in products that contained THC. More frequent use of these products in the previous 24 h was associated with improved reductions in pain intensity. These findings indicate a complex interrelationship between THC and CBD, with THC-associated reductions in daily pain intensity attenuated by increasing doses of CBD, and products containing both THC and CBD associated with longitudinal reductions in pain intensity. Statistically, the use of CBD-dominant products was not associated with any reductions in pain intensity at the daily or longitudinal levels, and these associations did not change regardless of how often participants used their products. Future research should assess these associations in larger samples to optimize THC and CBD doses and ratios to maximize both daily and longitudinal reductions in the pain associated with chronic low back pain.

## Figures and Tables

**Table 1:** Participant Characteristics.

	CBD-Dominant (n = 97)	THC + CBD (n = 112)	THC-Dominant (n = 34)	*p*-Value
**Demographics**				
**Age**	50.5 ± 14.5	45.8 ± 16.2	34.4 ± 13.6	<0.001
**Gender** (No. (%))				0.25
Female	58 (59.8)	64 (57.1)	13 (38.2)	
Male	37 (38.1)	46 (41.1)	20 (58.8)	
Non-Binary	2 (2.1)	2 (1.8)	1 (3.0)	
**Education** (No. (%) bachelor’s or higher)	69 (71.1)	82 (73.2)	16 (47.1)	0.01
**Race** (No. (%))				0.02
American Indian or Alaska Native	0 (0.0)	0 (0.0)	2 (5.9)	
Asian	1 (1.0)	2 (1.7)	1 (2.9)	
African American or Black	3 (3.1)	2 (1.7)	1 (2.9)	
Hispanic or Latino	1 (1.0)	7 (6.3)	1 (2.9)	
More than one race	5 (5.2)	6 (5.4)	5 (14.7)	
White	86 (88.7)	95 (84.8)	24 (70.6)	
Prefer not to answer	1 (1.0)	0 (0.0)	0 (0.0)	
**Baseline Descriptives**				
**Roland Morris Disability Questionnaire**	9.78 ± 5.03	9.03 ± 4.63	8.56 ± 4.49	0.34
**Pain Intensity**				
Current	3.27 ± 1.98	3.21 ± 1.97	3.59 ± 2.00	0.62
Average	4.44 ± 1.79	4.92 ± 1.66	4.88 ± 1.65	0.12
Worst	6.14 ± 2.02	6.78 ± 1.63	6.91 ± 1.16	0.01
**Cannabis Pain Expectancy**	2.12 ± 0.53	2.07 ± 0.55	2.14 ± 0.56	0.69
**Cannabis Use Disorder Symptoms**	0.20 ± 0.57	0.31 ± 0.99	0.53 ± 0.99	0.14
**Days of cannabis use**	0.43 ± 1.53	0.61 ± 1.17	1.41 ± 2.51	0.01
**Days of alcohol use**	3.63 ± 3.69	3.88 ± 3.79	3.38 ± 3.34	0.76
**Prescription Medications** (No. (%))	56 (57.7)	62 (55.4)	14 (41.2)	0.24

*Note. N* = 243. All data reported were collected during the baseline visit. Unless noted, results are reported as mean ± standard deviation. Scores for the Rolland Morris Disability Questionnaire range from 0–24, with higher scores indicating higher levels of disability due to low back pain. Current, average, and worst pain intensity were reported using a 0–10 scale, with “0” indicating no pain and “10” indicating the worst imaginable pain. Average and worst pain intensity were assessed in relation to the previous 7 days. Cannabis pain expectancy was measured using a 0–3 scale, with “0” indicating no improvement and “3” indicating very improved. Cannabis use disorder symptoms were measured using a DSM-5 modified Marijuana Dependence Scale. Substance use characteristics were measured via the Online Timeline Followback over the previous 14 days. Of the 132 participants who endorsed prescription medication, 15 (6.2%) endorsed opioids, 38 (15.6%) endorsed anti-depressants, 11 (4.5%) endorsed benzodiazepines, 8 (3.3%) endorsed beta blockers, 2 (0.8%) endorsed buspirone, 9 (3.7%) endorsed sleep medication, 12 (4.9%) endorsed migraine medication, 23 (9.5%) endorsed muscle relaxants, 12 (4.9%) endorsed non-steroidal anti-inflammatory (NSAID) medications, 19 (7.8%) endorsed nerve pain medication, 11 (4.5%) endorsed attention-deficit/hyperactivity disorder (ADHD) medication, and 60 (24.7) endorsed other medications [e.g., Suboxone, Quetiapine (Seroquel)].

**Table 2:** Cannabis Study Product Use.

	CBD-Dominant	THC + CBD	THC-Dominant
Days of study product use	9.38 ± 3.63	8.46 ± 3.91	7.65 ± 3.72
Total number of times participants used their product over the study	13.39 ± 7.76	11.04 ± 7.92	10.35 ± 8.55
Average number of times participants used their products on days of use	1.43 ± 0.80	1.30 ± 0.65	1.35 ± 0.78
THC Dose (mg)	2.27 ± 5.67	7.17 ± 8.07	16.85 ± 36.18
CBD Dose (mg)	26.19 ± 30.37	9.35 ± 11.35	5.90 ± 15.70

*Note.* Participant responses to the daily questionnaires were used to calculate days of study product use, total number of uses, average number of times they used their product on days of use, and delta-9-tetrahydrocannabinol (THC) and cannabidiol (CBD) dose. Participants were free to use their chosen recreational cannabis product as much or as little as they desired over the 14-day study period.

## Data Availability

Data will be made available upon request.
